# A live cell NanoBRET binding assay allows the study of ligand-binding kinetics to the adenosine A_3_ receptor

**DOI:** 10.1007/s11302-019-09650-9

**Published:** 2019-03-27

**Authors:** Monica Bouzo-Lorenzo, Leigh A. Stoddart, Lizi Xia, Adriaan P. IJzerman, Laura H. Heitman, Stephen J. Briddon, Stephen J. Hill

**Affiliations:** 1 Cell Signalling and Pharmacology Research Group, Division of Physiology, Pharmacology and Neuroscience, School of Life Sciences, University of Nottingham, Nottingham, UK; 2 Centre of Membrane Proteins and Receptors (COMPARE), University of Birmingham and University of Nottingham, Midlands, UK; 3 Division of Drug Discovery and Safety, Leiden Academic Centre for Drug Research, Leiden University, 9502, 2300 RA Leiden, The Netherlands

**Keywords:** Adenosine A_3_ receptor, Binding kinetics, Residence time, NanoBRET

## Abstract

There is a growing interest in understanding the binding kinetics of compounds that bind to G protein-coupled receptors prior to progressing a lead compound into clinical trials. The widely expressed adenosine A_3_ receptor (A_3_AR) has been implicated in a range of diseases including immune conditions, and compounds that aim to selectively target this receptor are currently under development for arthritis. Kinetic studies at the A_3_AR have been performed using a radiolabelled antagonist, but due to the kinetics of this probe, they have been carried out at 10 °C in membrane preparations. In this study, we have developed a live cell NanoBRET ligand binding assay using fluorescent A_3_AR antagonists to measure kinetic parameters of labelled and unlabelled compounds at the A_3_AR at physiological temperatures. The kinetic profiles of four fluorescent antagonists were determined in kinetic association assays, and it was found that XAC-ser-tyr-X-BY630 had the longest residence time (RT = 288 ± 62 min) at the A_3_AR. The association and dissociation rate constants of three antagonists PSB-11, compound 5, and LUF7565 were also determined using two fluorescent ligands (XAC-ser-tyr-X-BY630 or AV039, RT = 6.8 ± 0.8 min) as the labelled probe and compared to those obtained using a radiolabelled antagonist ([^3^H]PSB-11, RT = 44.6 ± 3.9 min). There was close agreement in the kinetic parameters measured with AV039 and [^3^H]PSB-11 but significant differences to those obtained using XAC-*S*-ser-*S*-tyr-X-BY630. These data indicate that selecting a probe with the appropriate kinetics is important to accurately determine the kinetics of unlabelled ligands with markedly different kinetic profiles.

## Introduction

The nucleoside adenosine is a ubiquitous signalling molecule which modulates cellular responses to stress. Upon cellular stress caused by mechanical, inflammatory or hypoxic stressors, high concentrations of ATP are released [1, 2] which are rapidly hydrolysed to adenosine [3]. Adenosine signals via four related family A G protein-coupled receptors (GPCRs) [4] of which the adenosine A_3_ receptor (A_3_AR) is thought to play an important role in the control of infection and related inflammation due to its expression on immune cells [5]. It has also been proposed to play a cardio- [6, 7] and neuro-protective [8, 9] role. In addition, the A_3_AR receptor has been shown to be expressed in a variety of cancer cell lines, and since the tumour micro-environment is often hypoxic, it may play a role in tumour progression [10] . This has led to an interest in developing molecules that target the A_3_AR in the treatment of immune conditions and cancer [11].

In recent years, many molecules targeting GPCRs, including those acting at adenosine receptors [12], have exhibited a lack of efficacy in clinical trials [13]. The optimization of molecules for clinical trials has historically focused on developing a candidate with high affinity and selectivity for the target receptor, and these parameters are often measured at equilibrium in model cell systems over-expressing the receptor of interest [14, 15]. Within the last 10 years, it has become clear that measuring the binding properties of a molecule at equilibrium may not be the most effective way to determine potential in vivo efficacy and that determining the duration of protein-drug interactions may be a better predictor of in vivo action [16, 17]. As the equilibrium dissociation constant (*K*_d_) of a ligand at a GPCR is a function of the ligand’s association (*k*_on_) and dissociation rate (*k*_off_) constants (*K*_d_ = *k*_off_/*k*_on_), molecules that have the same measured *K*_D_ at equilibrium can have markedly different *k*_on_ and *k*_off_ rate constants. To address this, Copeland et al. (2006) introduced the concept residence time (RT), which is the reciprocal of the dissociation rate constant (RT = 1/*k*_off_), as a measure of the duration of drug-target complex formation [18]. Depending on the clinical setting, different RTs may be required. For example, drugs with a long RT are preferred when an extended duration of action is required and can reduce administration to once a day. This has been demonstrated for the M3 muscarinic receptor antagonist tiotropium and the β_2_ adrenoceptor agonist olodaterol which both have long RTs and are once a day bronchodilators used to treat chronic obstructive pulmonary disease [14, 19, 20]. Short RT ligands are advantageous when the rapid, i.e. seconds to minutes, replacement of the drug by an endogenous ligand is crucial to avoid long-term side effects which has been proposed to be the case for drugs targeting the dopamine D_2_ receptor [17, 21, 22].

A variety of techniques exist to measure the kinetic parameters of molecules binding to the protein target of interest. As GPCRs are integral membrane proteins, biophysical techniques such as surface plasmon resonance which require purified protein can be challenging and often require mutagenesis to stabilise the receptor and allow it to be purified within lipid micelles or nanodiscs [23, 24]. Techniques that do not require the receptor to be purified include those that use radiolabelled or fluorescently labelled ligands [25, 26]. One limitation of these techniques is that they can only directly measure the kinetic parameters of the labelled compound. To overcome this, the methodology proposed by Motulsky and Mahan is often employed [27]. This technique measures the association kinetics of a labelled ligand in the presence of an unlabelled ligand and through knowledge of the kinetic parameters of the labelled ligand both the association and dissociation rate constants of the unlabelled ligand can be calculated. This technique is widely used with radiolabelled ligands and has more recently been successfully applied in combination with fluorescently labelled ligands in resonance energy transfer techniques such as time-resolved resonance energy transfer (TR-FRET) and bioluminescence resonance energy transfer (BRET) [28, 29].

For the A_3_AR, two recent studies have used radioligands to determine the kinetic parameters of unlabelled ligands [30, 31]. In the study by Xia et al., however, the radiolabelled antagonist ([^3^H]PSB-11) had to be used at low temperatures (10 °C) to slow the association rate sufficiently to give enough resolution to accurately determine the kinetic binding parameters of unlabelled ligands [30]. Due to this limitation and the inherent issue of throughput associated with radioligands [25], there is a need to develop additional methods to measure kinetic parameters. One way to potentially overcome these issues is through the use of fluorescently labelled ligand which have increased throughput, and previous studies have suggested that the fluorescent A_3_AR antagonist CA200645 has a slower association rate than [^3^H]PSB-11 [32]. For the A_3_AR, a number of fluorescently labelled antagonist probes have been developed based on two structurally distinct antagonists which have been shown to retain high affinity for the receptor [32–34]. Therefore, in this study, we have developed a live-cell BRET-based kinetic binding assay for the A_3_AR using four different fluorescently labelled antagonists. This assay has then been compared to the radioligand binding assay described in Xia et al. [30] for the determination of the kinetic binding parameters of unlabelled ligands.

## Material and methods

### Materials

Foetal calf serum (FCS) was obtained from PAA Laboratories (Wokingham, UK). Furimazine was purchased from Promega (Southampton, UK). Bicinchoninic acid protein assay kit and white 96-well microplates were obtained from Thermo Fisher Scientific (Waltham, MA, USA). GF/B filter plates and Microscint-O were from PerkinElmer (Groningen, The Netherlands). CA200645 was obtained from HelloBio (Bristol, UK). The synthesis of AV039 was described in Vernall et al. as compound 19 [34], while the synthesis of XAC-*S*-ser-*S*-tyr-X-BY630 (compound 27) and XAC-*S*-ser-*S*-tyr-X-BYFL (compound 28) was described in Vernall et al. 2013 [33]. PSB-11 and MRS1220 were purchased from Tocris Bioscience (Bristol, UK), and NECA was obtained from Sigma-Aldrich (Zwijndrecht, The Netherlands). [^3^H]8-Ethyl-4-methyl-2-phenyl-(8*R*)-4,5,7,8-tetrahydro-1*H*-imidazo[2,1-*i*]-purin-5-one ([^3^H]PSB-11) was kindly donated by Prof. C.E. Müller (University of Bonn, Germany) and its synthesis described in Müller et al. [35]. 1-Benzyl-8-methoxy-1H,3H-pyrido[2,1-f]purine-2,4-dione (compound 5) synthesis was described in Priego et al. as compound number 3 [36] and referred in Xia et al. [37] as compound number 5, while LUF7565 synthesis was described in Xia et al. as compound 27 [30]. All other chemicals and reagents were obtained from Sigma-Aldrich (Gillingham, UK).

### Cell culture and membrane preparation

Generation of human embryonic kidney 293 (HEK293) cells stably expressing the human A_3_AR tagged at the N-terminus with NanoLuc (Nluc-A_3_AR) is described in Stoddart et al. [38], and these cells were used throughout this study. Nluc-A_3_AR HEK293 cells were maintained in Dulbecco’s modified Eagle’s medium containing 10% FCS and 2 mM l-glutamine at 37 °C, 5% CO_2_.

For membrane preparation, Nluc-A_3_AR HEK293 cells were grown to confluence in 500-cm^2^ dishes. Normal growth media was replaced with ice-cold PBS, and the cells were removed from the dish using a cell scraper. The cells were then transferred to a 50-ml tube and centrifuged at 250×*g* for 5 min. The supernatant was discarded, and the resulting pellets were stored at − 80 °C. Thawed pellets were resuspended in ice-cold PBS and homogenised using an IKA T10 Ultra-Turrax disperser in 10 × 5 s bursts at 15,000 rpm. After removal of unbroken cells and nuclei by centrifugation at 1200×*g* for 10 min, the supernatant was centrifuged at 41,415×*g* for 30 min to obtain the membrane pellet. The pellet was then resuspended in ice-cold PBS and homogenised by 20 passes using a Kartell serrated pestle and a borosilicate glass homogeniser mortar fitted to an IKA RW16 overhead stirrer set to 1000 rpm. Finally, protein concentration was determined using a bicinchoninic acid protein assay [37] and membranes stored at − 80 °C until needed.

### NanoBRET binding assays

HEK293 cells stably expressing human Nluc-A_3_AR were seeded in normal growth medium 24 h prior to experimentation in white 96-well microplates coated with poly-d-lysine (100 μg/ml poly-d-lysine in PBS, 30 min room temperature, then washed in normal growth medium prior to use). Immediately before experimentation, media was replaced with HEPES buffered saline solution (HBSS; 145 mmol/L NaCL, 5 mmol/L KCl, 1.7 mmol/L CaCl_2_, 1 mmol/L MgSO_4_, 10 mmol/L HEPES, 2 mmol/L sodium pyruvate, 1.5 mmol/L NaHCO_3_, 10 mmol/L d-glucose, pH 7.4). For saturation and competition binding assays, the required concentration of fluorescent ligand and competing ligand was added simultaneously and incubated for 1 h (for AV039) or 3 h (for XAC-*S*-ser-*S*-tyr-X-BY630) at 37 °C (no CO_2_). After 1 h, 10 μM furimazine was added to each well and the plate incubated for 5 min at 37 °C before reading. Prior to all kinetic experiments, the medium was replaced by HBSS containing 10 μM furimazine and incubated at room temperature in the dark for 15 min to allow the luminescence signal to reach equilibrium [29]. For association kinetic experiments, following the furimazine incubation, the required concentration of fluorescent ligand in the presence or absence of 10 μM MRS1220 was added simultaneously and the plate read immediately once per minute for up to 3 h at 37 °C. For kinetic competition association assays, after furimazine incubation, either XAC-*S*-ser-*S*-tyr-X-BY630 (20 nM) or AV039 (40 nM) were added simultaneously with the required concentration of unlabelled ligand or 10 μM MRS1220 to determine non-specific binding and read once per minute for the indicated times at 37 °C. For all NanoBRET experiments, fluorescence and luminescence were read using a PHERAstar FS plate reader (BMG Labtech, Ortenberg, Germany). Filtered light emissions were measured at 460 nm (80 nm band pass) and at > 610 nm (long pass) for the BY630 labelled ligands and at 450 nm (80 nm band pass) and > 550 nm (long pass) for the BYFL labelled ligand. The raw NanoBRET ratio was calculated by dividing the fluorescence emission (610 or 550 nm) by the luminescence emission (460 or 450 nm).

### Radioligand binding assays

Prior to all experiments, membranes from HEK293 membranes expressing human Nluc-A_3_AR were diluted to 20 μg/well in a total volume of 100 μL/well assay buffer (50 mM Tris-HCl, 5 mM MgCl_2_, supplemented with 0.01% CHAPS and 1 mM EDTA, pH 7.4) and homogenised using an IKA T10 Ultra-Turrax disperser in 3 × 5 s bursts at 15,000 rpm. For equilibrium displacement assays, membranes were placed in 96-well microplates at 10 °C and the required concentration of competing agonist was added in the presence of a final concentration of ~ 10 nM [^3^H] PSB-11 with nonspecific binding determined in the presence of 100 μM NECA (final concentration). For association assays, Nluc-A_3_AR HEK293 membranes were placed at 10 °C in 96-well microplates and the amount of radioligand bound to the receptor was measured at different time points during a total incubation of 120 min. For dissociation experiments, HEK293 membranes expressing human Nluc-A_3_AR were incubated for 120 min with ~ 10 nM [^3^H] PSB-11 at 10 °C prior the addition of 10 μM PSB-11 (final concentration) at various time points during a further 120 min. The competition association assays were initiated by the addition of Nluc-A_3_AR HEK293 membranes at different time points for a total of 240 min to a total volume of 100 μl/well of assay buffer at 10 °C with ~ 10 nM [^3^H] PSB-11 in the absence or presence of a single concentration (2 × *K*_i_) of competing A_3_AR ligands. For all experiments, incubation was terminated by rapid filtration performed on 96-well GF/B filter plates using a PerkinElmer Filtermate-harvester (PerkinElmer, Groningen, The Netherlands). After drying the filter plate at 50 °C for 30 min, the filter-bound radioactivity was determined by scintillation spectrometry using a 2450 MicroBeta^2^ Plate Counter (PerkinElmer, Boston, MA). In addition, the exact concentration of [^3^H] PSB-11 used in each experiment was determined by scintillation spectrometry and this concentration was used in the data analysis.

### Data analysis

All experimental data were analysed using Prism7 (GraphPad Software, San Diego, CA).

NanoBRET total and non-specific saturation binding curves were fitted simultaneously using the following equation:

$$ \mathrm{BRET}\ \mathrm{ratio}=\frac{B_{\mathrm{max}}.\left[B\right]}{\left[B\right]+{K}_{\mathrm{D}}}+\left(\left(M.\left[B\right]\right)+C\right) $$

where *B*_max_ is the maximal specific binding achieved, [*B*] is the concentration of fluorescent ligand, *K*_D_ is the equilibrium dissociation constant, *M* is the slope of the non-specific binding component and *C* is the intercept with the *Y*-axis.

Equilibrium competition binding curves were fitted with the following equation:

$$ {K}_{\mathrm{i}}=\frac{{\mathrm{IC}}_{50}}{1+\frac{\left[L\right]}{K_{\mathrm{D}}}} $$

where [*L*] is the concentration of [^3^H]PSB11, AV039 or XAC-*S*-ser-*S*-tyr-X-BY630 and *K*_D_ is the equilibrium dissociation constant of the labelled ligand (9.9 nM for [^3^H]PSB-11, 14.5 nM for XAC-*S*-ser-*S*-tyr-X-BY630 and 32.5 nM for AV039 as determined in saturation binding assay performed as part of this study). The IC_50_ is obtained as follows:

$$ \%\mathrm{inhibition}\ \mathrm{of}\ \mathrm{specific}\ \mathrm{binding}=\frac{100.\left[A\right]}{\left[A\right]+{\mathrm{IC}}_{50}} $$

where [*A*] is the concentration of unlabelled competing drug and IC_50_ is the molar concentration of this competing ligand required to inhibit 50% of the specific binding of the concentration [*L*] of the labelled ligand.

For NanoBRET association kinetic data, non-specific binding was determined in wells with fluorescent ligand plus 10 μM MRS1220 and this was obtained for each concentration of fluorescent ligand at each time point. This was subsequently subtracted from total binding at the equivalent time point. The data were simultaneously fit to the following equations:

$$ Y={Y}_{\mathrm{max}}\left(1-{e}^{-{k}_{\mathrm{obs}}t}\right) $$

$$ {k}_{\mathrm{on}}=\frac{k_{\mathrm{obs}}-{k}_{\mathrm{off}}}{\left[L\right]} $$

where *Y*_max_ equals the level of binding at infinite time (*t*), *k*_obs_ is the rate constant for the observed rate of association at a particular concentration of *L*, [*L*] is the ligand concentration in molar, *k*_off_ is the dissociation rate constant of the ligand in per minute and *k*_on_ is the association rate constant in per molar per minute. From this, the equilibrium dissociation constant (K_D_) is determined as follows:

$$ {K}_{\mathrm{D}}=\frac{k_{\mathrm{off}}}{k_{\mathrm{on}}} $$

The binding kinetics of unlabelled ligands was quantified using the competition association assay based on the theoretical framework by Motulsky and Mahan [27]. NanoBRET and radioligand data were fitted into the competition association model using ‘kinetics of competitive binding’ to determine association and dissociation rate constants of the unlabelled compounds:

$$ {\displaystyle \begin{array}{c}\begin{array}{c}{K}_A={k}_1\left[L\right]\cdot {10}^{-9}+{k}_2\\ {}{K}_B={k}_3\left[I\right]\cdot {10}^{-9}+{k}_4\;\\ {}S=\sqrt{{\left({K}_A-{K}_B\right)}^2+4\cdot {k}_1\cdot {k}_3\cdot L\cdot I\cdot {10}^{-18}}\end{array}\\ {}{K}_F=0.5\left({K}_A+{K}_B+S\right)\\ {}{K}_S=0.5\left({K}_A+{K}_B-S\right)\\ {}\begin{array}{c}\begin{array}{c}Q=\frac{B_{\mathrm{max}}\cdot {k}_1\cdot L\cdot {10}^{-9}}{K_F-{K}_S}\\ {}Y=Q\cdot \frac{k_4\cdot \left({K}_F-{K}_S\right)}{K_F\cdot {K}_S}+\frac{k_4-{K}_F}{K_F}{e}^{\left(-{K}_F\cdot X\right)}-\frac{k_4-{K}_S}{K_S}{e}^{\left(-{K}_S\cdot X\right)}\end{array}\\ {}\end{array}\end{array}} $$

where *k*_1_ is the *k*_on_ of the labelled ligand (M^−1^ min^−1^), *k*_2_ is the *k*_off_ of the labelled ligand (min^−1^), *L* is the concentration of the labelled ligand in nanomolar, *I* is the concentration of the unlabelled competitor (nM), *X* is the time (min) and *Y* is the specific binding of the labelled ligand (NanoBRET ratio or DPM).

*k*_1_ and *k*_2_ values were generated from association kinetic experiments. *k*_3_, *k*_4_ and *B*_max_ were then calculated, where *k*_3_ represents the *k*_on_ (M^−1^ min^−1^) of the unlabelled ligand, *k*_4_ stands for the *k*_off_ (min^−1^) of the unlabelled ligand and *B*_max_ equals the total binding (NanoBRET ratio or DPM). The residence time (RT) was calculated as the reciprocal of the ligand dissociation rate constant as follows:

$$ \mathrm{RT}=\frac{1}{k_{\mathrm{off}}} $$

Statistical significance was determined by Student’s unpaired *t* test where *p* < 0.05 was considered statistically significant throughout this study.

## Results

### NanoBRET binding profile of fluorescent probes at Nluc-A_3_AR in live cells

**Fig. 1:**
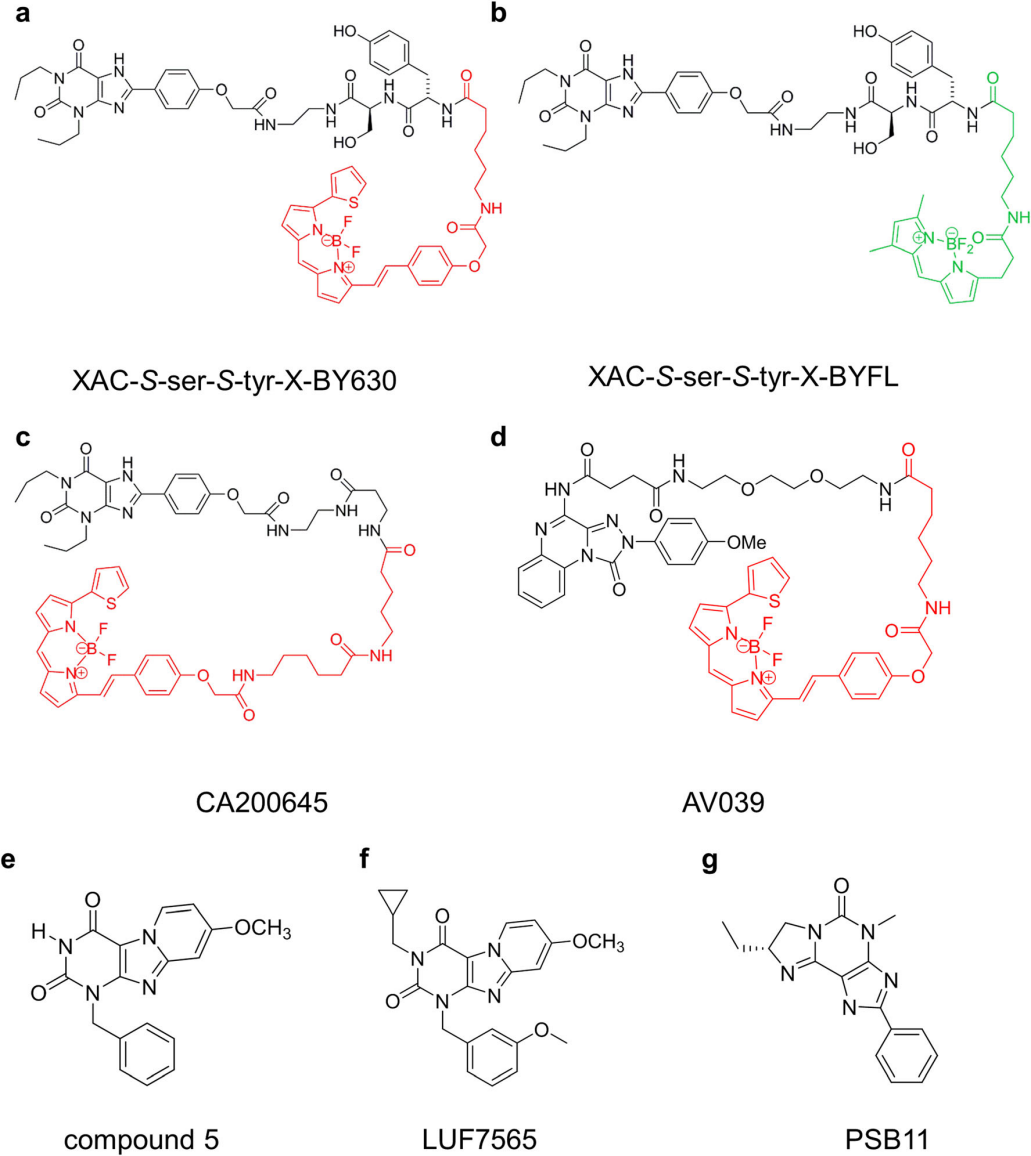
Structures of the A_3_AR fluorescent (**a**–**d**) and unlabelled (**e**–**g**) antagonists used in this study

The four fluorescent ligands used in this study have previously been shown to display high affinity for the A_3_AR, and their structures are shown in Fig. 1 [32–34]. Three of the ligands are based on the non-selective adenosine receptor antagonist xanthine amine congener (XAC). Two of these XAC linked compounds contain a serine, tyrosine dipeptide (both in the *S* configuration) to link the pharmacophore to the fluorophore which is either BODIPY630/650 (excitation max 630 nm, emission max 650 nm) to give XAC-*S*-ser-*S*-tyr-BY630 (Fig. 1a, or BODIPY-FL (excitation max 503 nm, emission max 512 nm) to give XAC-*S*-ser-*S*-tyr-BYFL (Fig. 1b) [33]. The third XAC-based compound, CA200645 (Fig. 1c), contains a polyamide linker (β-alanine, β-alanine) connected to the BY630 fluorophore [32]. AV039 (Fig. 1d) is based on the A_3_AR selective antagonist 1,2,4-triazolo[4,3-a]quinoxalin-1-one linked to BY630 and has been shown to retain A_3_AR selectivity [34].

**Fig. 2:**
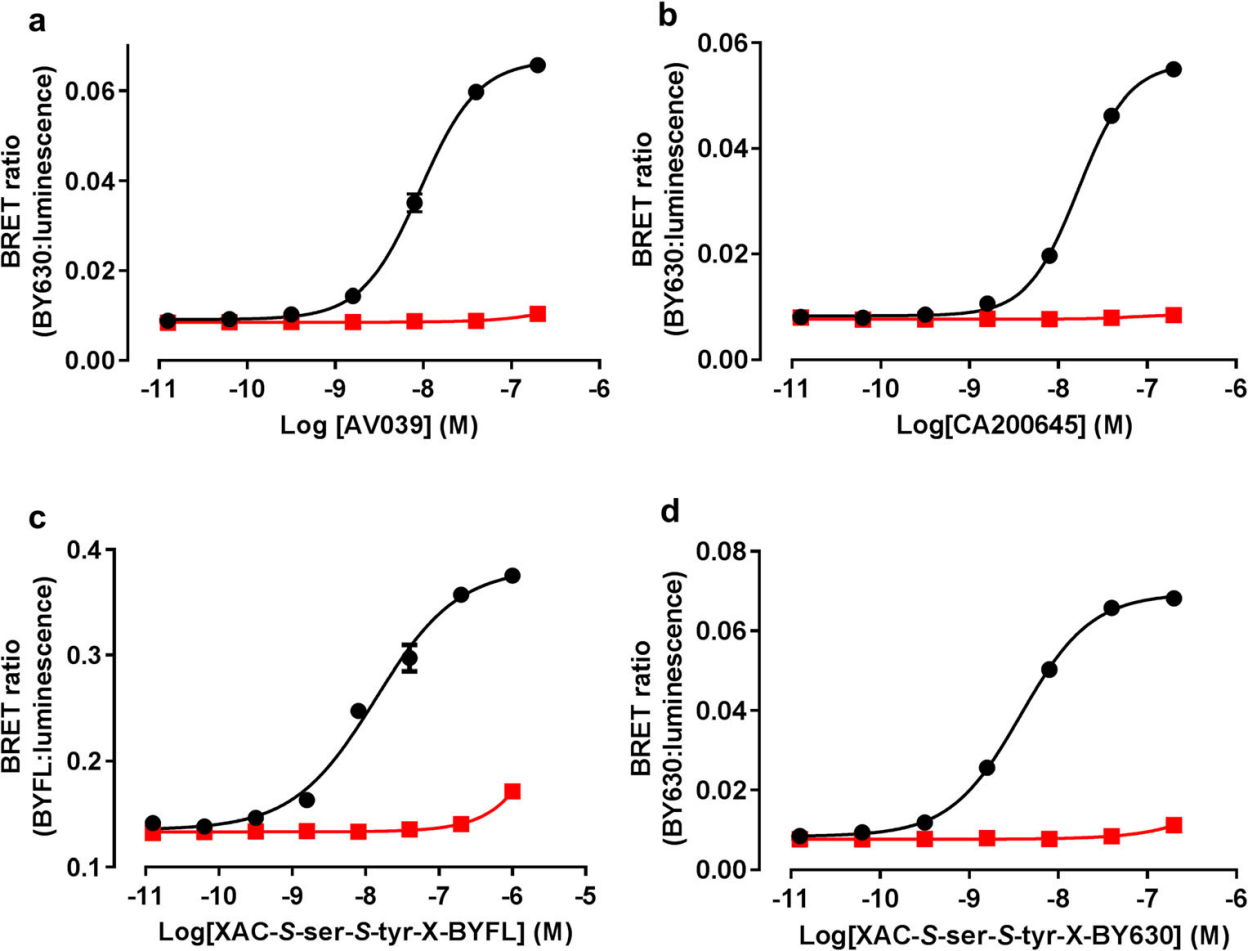
Determination of the binding affinity of four fluorescent antagonists at human A_3_AR. NanoBRET saturation binding curves obtained by treating Nluc-A_3_AR HEK293 cells with increasing concentrations of the fluorescent antagonists AV039 (**a**), CA200645 (**b**), XAC-*S*-ser-*S*-tyr-X-BYFL (**c**) or XAC-*S*-ser-*S*-tyr-X-BY630 (**d**) in the absence (black circles) or presence (red squares) of 10 μM MRS1220 for 1 h at 37 °C. The data shown are mean ± SEM and are representative of five independent experiments each performed in triplicate

Initially, we determined the affinity of each of these fluorescent antagonists at NanoLuc tagged A_3_AR (Nluc-A_3_AR) in the NanoBRET assay. An advantage of NanoBRET compared to radioligand binding experiments is the large concentration range of fluorescent ligands over which binding can be measured, and to represent this accurately, binding is fitted as a sigmoidal curve with BRET ratio values plotted versus the log concentration of fluorescent ligand. In all the cases, we obtained a saturable curve clearly dependent on the concentration of the fluorescent ligands (Fig. 2). Non-specific binding was determined by co-incubation with 10 μM of the unlabelled high affinity A_3_AR antagonist MRS1220, and all four fluorescent ligands exhibited very low levels of non-specific NanoBRET in line with previously published NanoBRET data [29, 38]. The point on the curve which gives the 50% of the binding was then taken as *K*_D_. The pK_D_ values calculated for the four fluorescent antagonists are shown in Table 1. All four ligands displayed high affinities for Nluc-A_3_AR which were consistent with literature values, and the range of pK_D_ values were found to span less than 1 log unit (pK_D_ 7.78–8.11).

**Table 1:** Kinetic parameters and binding affinities of fluorescent antagonists at the human A_3_AR measured by NanoBRET

	Kinetics					Equilibrium saturation	
Compound	pK_D_	*k*_on_ (× 10^6^) (M^−1^ min^−1^)	*k*_off_ (min^−1^)	RT (min)	*n*	pK_D_	*n*
AV039	7.35 ± 0.10*	3.67 ± 0.62	0.15 ± 0.02	6.8 ± 0.8	4	8.11 ± 0.10*	5
CA200645	7.58 ± 0.04*	2.58 ± 0.05	0.069 ± 0.006	14.8 ± 1.1	5	7.80 ± 0.07*	5
XAC-*S*-ser-*S*-tyr-X-BY630	8.57 ± 0.15	1.66 ± 0.36	0.0043 ± 0.0009	288 ± 62	5	8.51 ± 0.07	5
XAC-*S*-ser-*S*-tyr-X-BYFL	7.75 ± 0.14	3.95 ± 0.73	0.069 ± 0.006	14.9 ± 1.1	4	7.78 ± 0.05	5

Saturation pK_D_ values were calculated directly from the saturation binding curves of the fluorescent ligands binding to Nluc-A_3_AR HEK293 cells. The kinetic parameters, *k*_on_, *k*_off_ and pK_D_ values, were obtained by monitoring the NanoBRET signal over time of various concentrations of fluorescent ligand in HEK293 Nluc-A_3_AR cells at 37 °C. The residence time (RT) was calculated as the reciprocal of the *k*_off_ values from each individual experiment. All values represent mean ± SEM from *n* separate experiments performed in triplicate

*p < 0.05, kinetic versus equilibrium saturation pK_D_ values according to unpaired Student’s *t* test

**Fig. 3:**
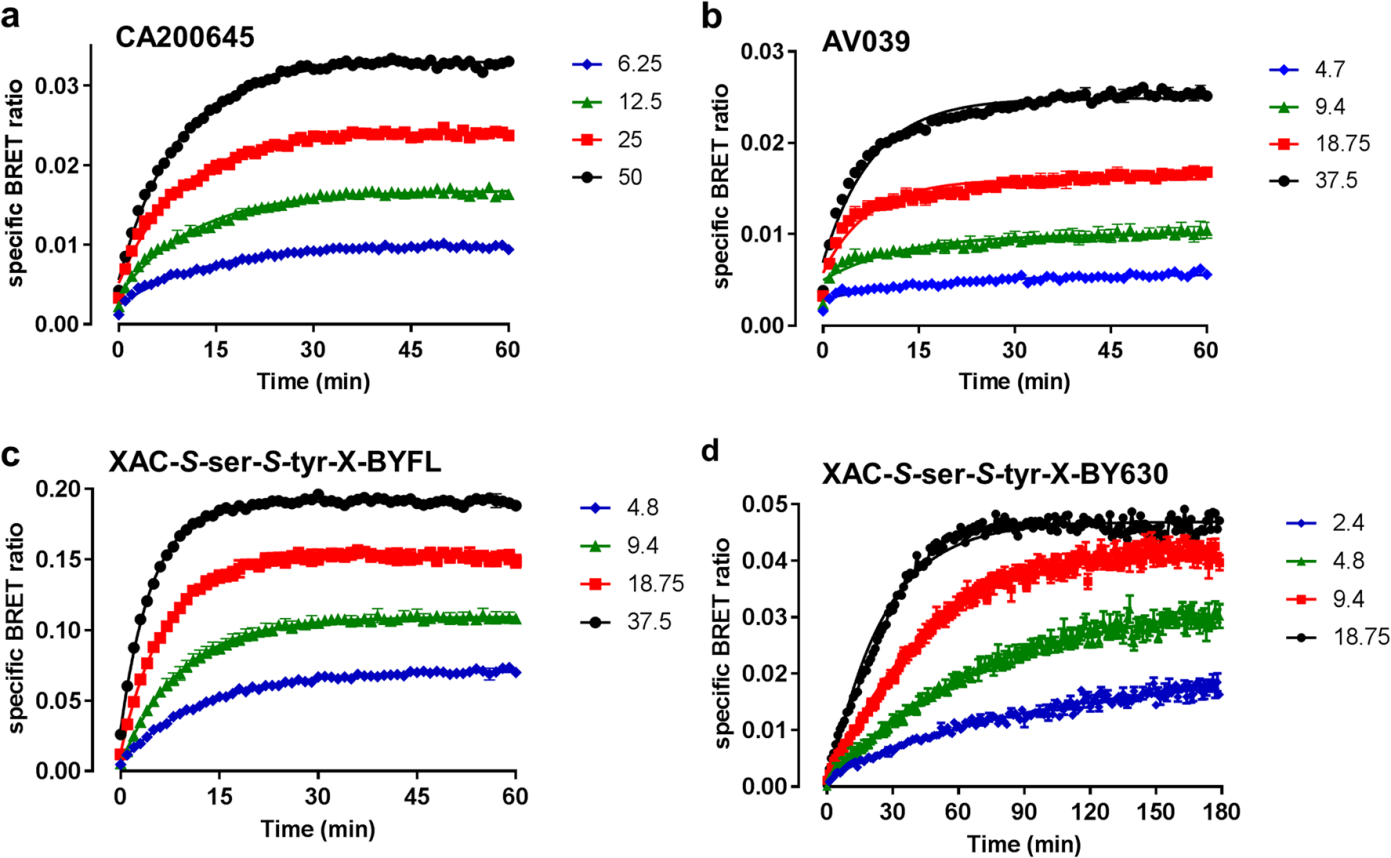
Association kinetics of four fluorescent antagonists at human A_3_AR. After 15 min pre-incubation with 0.5 μM furimazine, Nluc-A_3_AR HEK293 cells were treated with the indicated concentrations of CA200645 (**a**), AV039 (**b**), XAC-*S*-ser-*S*-tyr-X-BYFL (**c**) or XAC-*S*-ser-*S*-tyr-X-BY630 (**d**) and the NanoBRET signal was monitored at 37 °C every min for 60 min (**a**–**c**) or 180 min (**d**). The data shown are mean ± SEM and are representative examples from four (**a**, **c**) or five (**b**, **d**) independent experiments, each performed in triplicate

To determine the kinetic parameters of fluorescent ligands at the A_3_AR, Nluc-A_3_AR expressing HEK293 cells were initially treated with the Nanoluc substrate, furimazine (10 μM), for 15 min prior to the fluorescent ligand to allow the luminescence signal to stabilise as described previously [29]. Increasing concentrations of fluorescent ligands were then added and the change in NanoBRET monitored at 37 °C over time, with specific binding determined in the presence of the high affinity A_3_AR antagonist MRS1220 (10 μM) for every time point (Fig. 3). The kinetic parameters obtained from globally fitting the association curves showed that all four fluorescent ligands had similar association rates within a factor of two of each other (Table 1). As expected, the range of dissociation rates for AV039, CA200645 and XAC-*S*-ser-*S*-tyr-X-BYFL were also within a factor of two of each other, leading to similar RTs. The dissociation rate for XAC-*S*-ser-*S*-tyr-X-BY630, however, was over ten times slower than that of the other three fluorescent ligands. This produced a markedly different RT for XAC-*S*-ser-*S*-tyr-X-BY630 which was of the order of hours (4.8 h) compared to the much more rapid values (min) obtained for the other three compounds. Additionally, it can be noted that the pK_D_ values from saturation studies of XAC-*S*-ser-*S*-tyr-X-BY630 and XAC-*S*-ser-*S*-tyr-X-BYFL are comparable to those obtained from the kinetic data, whereas for CA200645 and AV039, the saturation pK_D_ values are significantly higher than the kinetic pK_D_ (Table 1, *p* < 0.05, unpaired *t* test).

### Radioligand binding characterisation of fluorescent probe

**Fig. 4:**
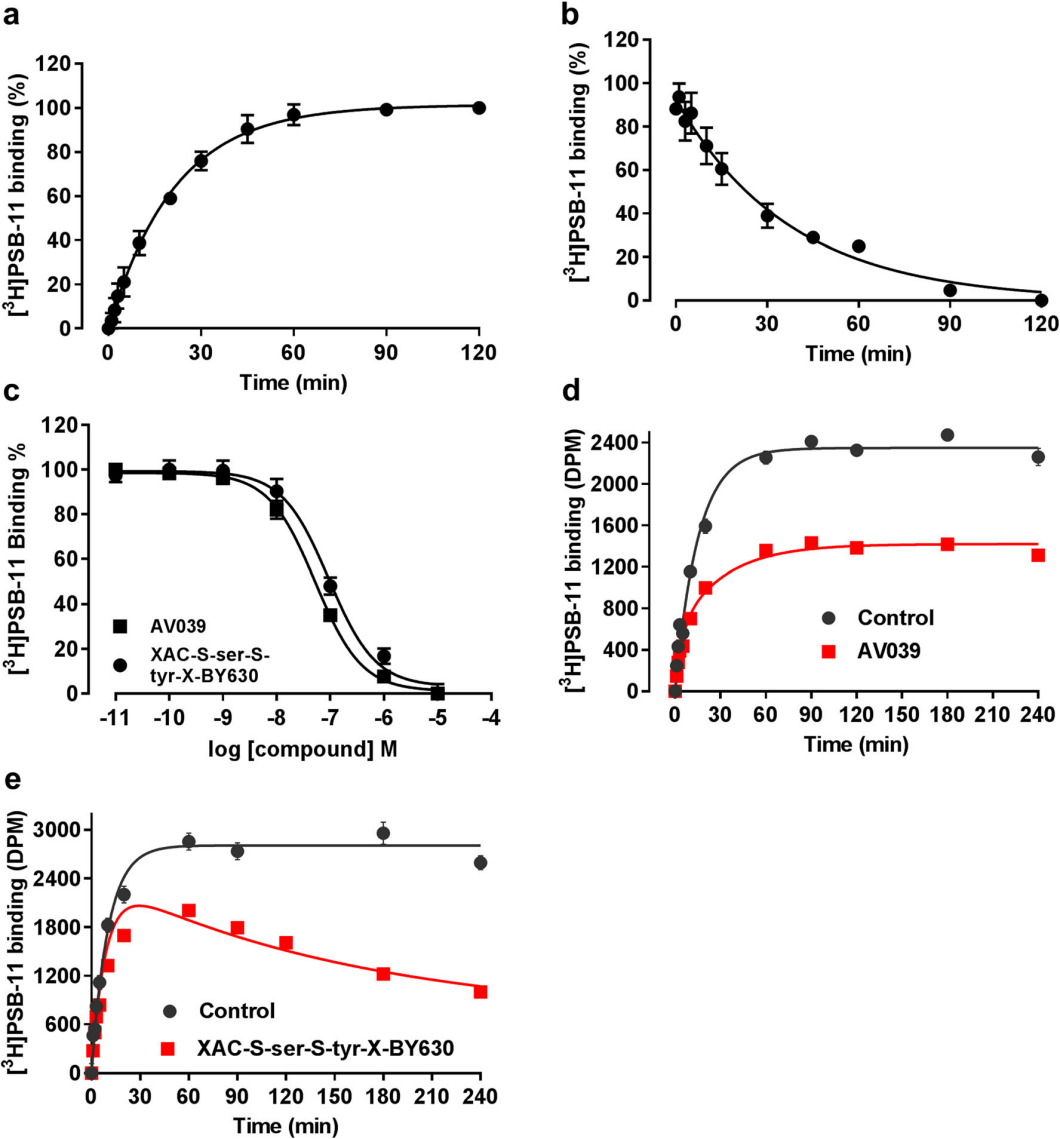
Radioligand binding characterisation of fluorescent probes at human A_3_AR. Association (**a**) and dissociation (**b**) curves of 10 nM [^3^H]PSB-11 in Nluc-A_3_AR HEK293 membranes performed at 10 °C. Dissociation of the radioligand was initiated by addition of 10 μM PSB-11 after equilibrium had been reached at 2 h. Data were normalised to the maximal [^3^H]PSB-11 labelling in each experiment and shown as the mean and S.E.M. of five independent experiments performed in duplicate. **c** Nluc-A_3_AR membranes were treated with increasing concentrations of AV039 (squares) and XAC-*S*-ser-*S*-tyr-X-BY630 (circles) and 10 nM [^3^H]PSB-11 in HEK293 Nluc-A_3_AR membranes at 10 °C to generate competition binding curves. Data were normalised to the maximal specific [^3^H]PSB-11 labelling in each experiment. Data points are combined mean ± SEM from five separate experiments performed in duplicate. **d**, **e** Competition association time course of ~ 10 nM [^3^H]PSB-11 on HEK293 Nluc-A_3_AR membranes at 10 °C in either absence (control, black circles) or presence of a single concentration (2 × *K*_i_ value) of AV039 (**d**, red squares) or XAC-*S*-ser-*S*-tyr-X-BY630 (**e**, red squares). The data shown are representative examples from four (**d**) and five (**e**) independent experiments performed in duplicate with each data point shown

To confirm the differences in the kinetics observed with AV039 and XAC-*S*-ser-*S*-tyr-X-BY630, radioligand equilibrium and kinetic binding assays in membranes from HEK293 cells stably expressing Nluc-A_3_AR were undertaken. Initially, the binding affinity and kinetic parameters of the radiolabelled A_3_AR antagonist [^3^H]PSB-11 at Nluc-A_3_AR were resolved from its association and dissociation curves at 10 °C (Fig. 4a, b; Table 2) and were similar to those obtained previously with the wild-type A_3_AR [30]. Next, the ability of XAC-*S*-ser-*S*-tyr-X-BY630 and AV039 to compete at equilibrium with specific [^3^H]PSB-11 binding to the Nluc-A_3_AR was examined (Fig. 4c) and the pKi for each of these compounds determined (Table 2). The affinities obtained in the radioligand binding assay for both fluorescent probes were lower than those obtained in the NanoBRET experiments (Table 1), which may reflect differences in affinity values determined in whole cells compared to cell membranes, and also the impact of temperature (10 °C versus 37 °C), as previously observed for the histamine H_1_ receptor [29].

**Table 2:** Radioligand binding affinities and kinetic parameters of fluorescent probes and unlabelled ligands at the human A_3_AR

	Kinetics					Equilibrium competition	
	pK_D_	*k*_on_ (× 10^6^ M^−1^ min^−1^)	*k*_off_ (min^−1^)	RT (min)	*n*	pK_i_	*n*
[^3^H]PSB-11	8.04 ± 0.08	2.75 ± 0.52	0.023 ± 0.002	44.6 ± 3.9	5	ND	ND
AV039	7.63 ± 0.08	3.27 ± 0.47	0.076 ± 0.009	14.0 ± 1.6	5	7.65 ± 0.02	5
XAC-*S*-ser-*S*-tyr-X-BY630	7.74 ± 0.08*	0.14 ± 0.04	0.0023 ± 0.0002	447 ± 44	4	7.21 ± 0.06*	5
compound 5	7.53 ± 0.13	6.61 ± 1.76	0.198 ± 0.045	6.5 ± 2.1	4	7.35 ± 0.02	5
LUF7565	9.11 ± 0.04*	8.17 ± 0.95	0.0065 ± 0.0014	168 ± 25	4	8.99 ± 0.02*	5

pK_i_ values were calculated from inhibition of equilibrium [^3^H]PSB-11 binding to Nluc-A_3_AR HEK293 membranes at 10 °C. The kinetic parameters, *k*_on_, *k*_off_ and pK_D_ values of PSB-11 were obtained from association and dissociation curves of 10 nM [^3^H]PSB-11 in Nluc-A_3_AR HEK293 membranes at 10 °C. Radioligand dissociation was initiated by the addition of 10 μM unlabeled PSB-11. The kinetic parameters, *k*_on_, *k*_off_ and pK_D_ values of AV039, XAC-*S*-ser-*S*-tyr-X-BY630, compound 5 and LUF7565 were obtained by competition association with ~ 10 nM [^3^H]PSB-11 at 10 °C. The RT was calculated as the reciprocal of the *k*_off_ values from each individual experiment. All values represent mean ± SEM from *n* separate experiments performed in duplicate

*ND* not determined

*p < 0.05, kinetic pK_D_ versus equilibrium competition pK_i_ values according to unpaired Student’s *t* test

The association and dissociation rates of XAC-*S*-ser-*S*-tyr-X-BY630 and AV039 were determined in kinetic association assays where the specific binding of [^3^H]PSB-11 in the presence and absence of a single concentration (2 × IC_50_) of the fluorescent ligand was monitored over time and the resulting data fitted to the model proposed by Motulsky and Mahan [27]. As expected from the kinetic behaviour observed in the NanoBRET assay, the association curve of [^3^H]PSB-11 obtained in the presence of AV039 quickly reached an equilibrium plateau (Fig. 4d) indicating that AV039 has a similar or faster kinetic profile compared to [^3^H]PSB-11. This was borne out in the kinetics parameters obtained from fitting the data to the Motulsky and Mahan model (Table 2). In comparison, XAC-*S*-ser-*S*-tyr-X-BY630 induced an ‘overshoot’ in [^3^H]PSB-11 specific binding followed by a steady decrease (Fig. 4e) which is characteristic of long RT compounds. As such, both the association and dissociation rate constants of this fluorescent ligand are more than 10 times slower than those of AV039, whilst the pK_D_ values are very similar. As a consequence, it was confirmed that XAC-*S*-ser-*S*-tyr-X-BY630 had a long RT (over 7.5 h) compared to AV039 and [^3^H]PSB-11.

### Determination of the affinity and kinetic constants of unlabelled ligands

**Fig. 5:**
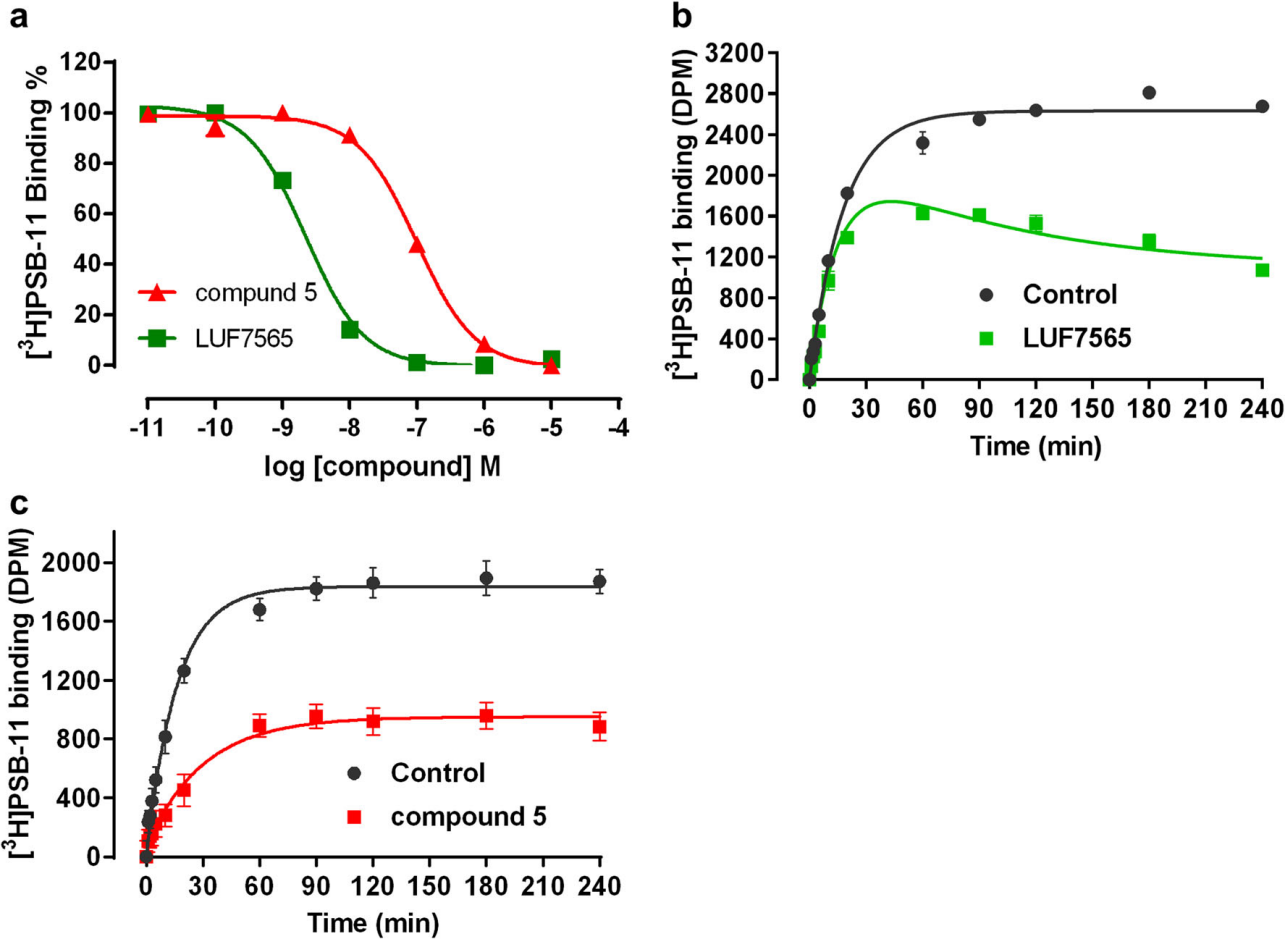
Radioligand binding characterisation of unlabelled ligands at human A_3_AR. **a** HEK293 membranes expressing human Nluc-A_3_AR were treated with increasing concentrations of compound 5 (red triangles) and LUF7565 (green squares), and 10 nM [^3^H]PSB-11 at 10 °C for 2 h and levels of [^3^H]PSB-11 binding monitored by scintillation counting. Data were normalised to the maximal specific [^3^H]PSB-11 labelling in each experiment. Data points are combined mean ± SEM from five separate experiments performed in duplicate. **b**, **c** Association of [^3^H]PSB-11 on Nluc-A_3_AR HEK293 membranes at 10 °C in the absence (control, black circles) or presence of a single concentration (2 × *K*_i_ value) of and LUF7565 (**b**) or compound 5 (**c**). The data shown are representative examples from four independent experiments performed in duplicate with each data point shown

To extend the use of the NanoBRET binding assay, it is necessary to use this setup to determine the kinetics of unlabelled ligands using the Motulsky and Mahan approach. To demonstrate its utility, it is important that the NanoBRET assay can distinguish between fast and slow unlabelled compounds. First, it was important to select unlabelled compounds with the desired kinetic profile. Using [^3^H]PSB-11 as the tracer, the kinetic parameters of two previously described high-affinity A_3_AR antagonists, compound 5 [36] (Fig. 1e) and LUF7565 [30] (Fig. 1f) were determined at Nluc-A_3_AR. Competition binding experiments indicated compound 5 and LUF7565 gave a concentration-dependent inhibition of the specific binding of [^3^H]PSB-11 with LUF7565 displaying higher affinity for Nluc-A_3_AR than compound 5 (Fig. 5a; Table 2). The association and dissociation rate constants of compound 5 and LUF7565 were then determined from the decrease in the specific binding of [^3^H]PSB-11 in the presence of a single concentration (2 × IC_50_) of the unlabelled ligands. The competition association curve of compound 5 (Fig. 5c) exhibited the kinetic profile of a short RT ligand, while LUF7565 induced an ‘overshoot’ in the [^3^H]PSB-11-specific binding (Fig. 5b) which is the characteristic profile of a long RT compound, and this was confirmed from the calculated kinetic parameters (Table 2). Radioligand kinetic affinity was comparable to that obtained by radioligand displacement for both the unlabelled ligands.

**Fig. 6:**
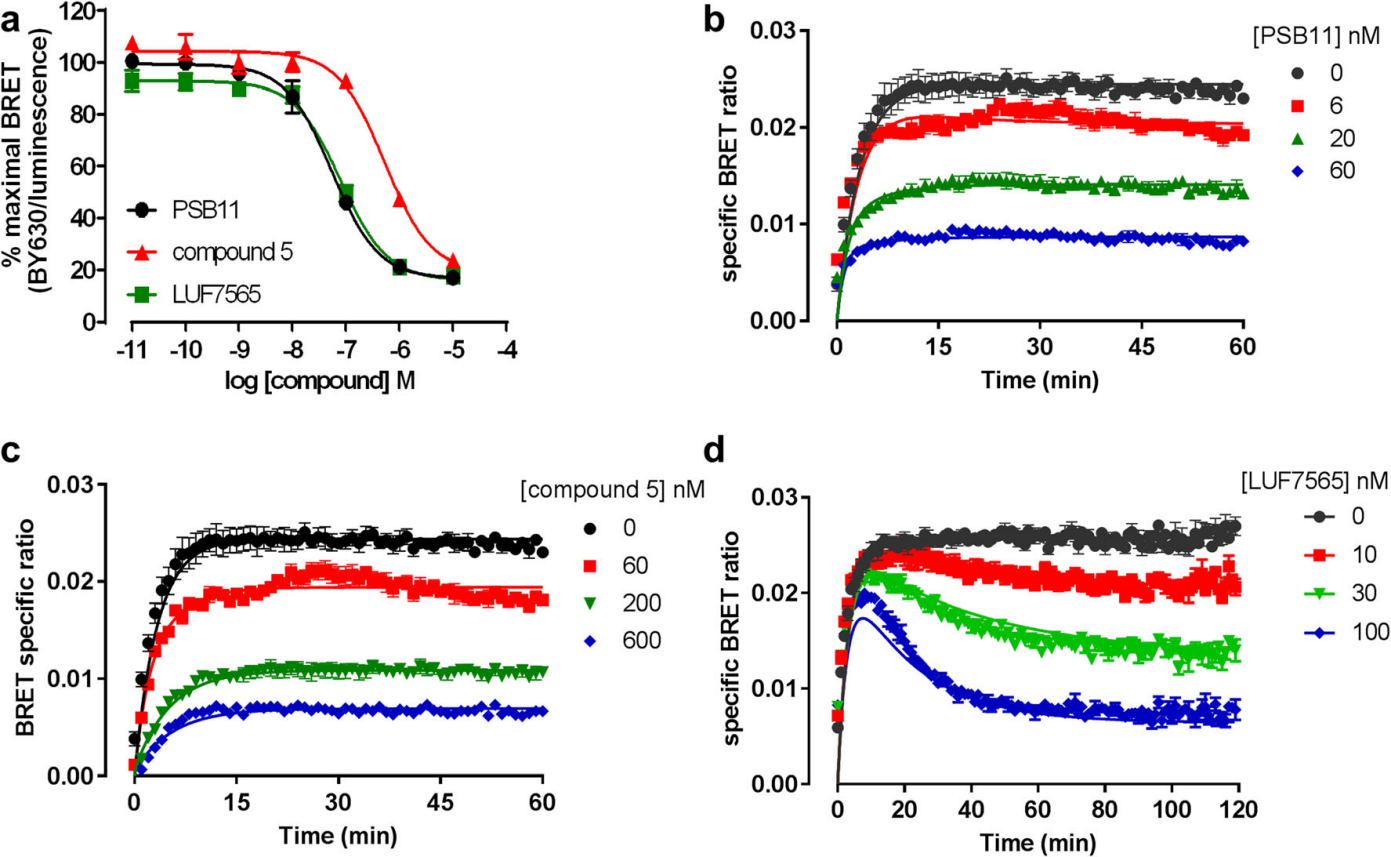
Characterisation of the effect of unlabelled ligands on the binding of AV039 to Nluc-A_3_AR using NanoBRET (**a**). Nluc-A_3_AR cells were treated with 50 nM AV039 and increasing concentrations of PSB-11 (black circles), compound 5 (red triangles) and LUF7565 (green squares) for 1 h at 37 °C and competition binding curves generated. Data were normalised to the maximal BRET signal in each experiment. Data points are combined mean ± SEM from five separate experiments performed in triplicate. **b**–**d** After 15 min pre-incubation with 0.5 μM furimazine, the association rate of 40 nM AV039 was monitored, via NanoBRET, at 37 °C in HEK293 cells expressing human Nluc-A_3_AR in the absence (control, black circles) or presence of the indicated concentrations of PSB-11 (**b**), compound 5 (**c**) and LUF7565 (**d**). The data shown are representative examples from five (**b**, **c**) or six (**d**) independent experiments performed in triplicate, and the depicted data points represent the mean ± SEM of the triplicates

The selective A_3_AR fluorescent antagonist AV039 was chosen as a short RT probe to evaluate the affinity and kinetic parameters of compound 5, LUF7565 alongside those of PSB-11. Initially, the affinity of these three antagonists was determined in an equilibrium competition assay using 20 nM AV039 in HEK 293 cells stably expressing the Nluc-A_3_AR (Fig. 6a; Table 3). As expected, all three ligands exhibited high affinity for the A_3_AR, confirming the data obtained using [^3^H]PSB-11. To further investigate the properties of these compounds, their binding kinetic parameters were determined using AV039 as the probe. The time-dependent decrease in the association binding of AV039 induced by the addition of different concentrations of PSB-11, compound 5 and LUF7565 was monitored at 37 °C (Fig. 6b–d) to determine the kinetic parameters of these unlabelled compounds. The competition association curves of PSB-11 (Fig. 6b) and compound 5 (Fig. 6c) exhibited the typical kinetic profile of a short RT ligand, while LUF7565 (Fig. 6d) induced a very pronounced initial overshoot in binding of AV039 followed by a decrease which is characteristic of a long RT compound. As expected from the shape of the competition association curves, LUF7565 was found to have the slowest dissociation rate and subsequently the longest RT (Table 3). The kinetic pK_D_ of the short RT compounds PSB-11 and compound 5 was similar to the equilibrium pK_i_, while for the slow dissociation compound LUF7565, the kinetic pK_D_ was significantly higher than pK_i_ (*p* < 0.05, unpaired *t* test).

**Table 3:** Binding affinities and kinetic parameters of unlabelled ligands at the human A_3_AR measured by NanoBRET

Unlabelled ligand	AV039							XAC-*S*-ser-*S*-tyr-X-BY630						
	Kinetics					Equilibirum competition		Kinetics					Equilibrium competition	
	pK_D_	*k*_on_ (× 10^6^) (M^−1^ min^−1^)	*k*_off_ (min^−1^)	RT (min)	*n*	pK_i_	*n*	pK_D_	*k*_on_ (× 10^6^) (M^−1^ min^−1^)	*k*_off_ (min^−1^)	RT (min)	*n*	pK_i_	*n*
PSB-11	7.83 ± 0.05*	11.7 ± 1.4	0.18 ± 0.04	6.6 ± 1.3	5	8.09 ± 0.05*	5	7.48 ± 0.09	1.59 ± 0.33	0.053 ± 0.010	22.4 ± 6.1	4	7.20 ± 0.07	3
Compound 5	6.91 ± 0.05	11.0 ± 3.2	1.41 ± 0.44	1.1 ± 0.4	5	7.05 ± 0.04	5	6.54 ± 0.02*	0.16 ± 0.02	0.047 ± 0.009	23.2 ± 3.6	4	6.08 ± 0.09*	3
LUF7565	8.17 ± 0.12	2.68 ± 0.78	0.015 ± 0.002	72.9 ± 8.3	6	7.93 ± 0.07	5	7.87 ± 0.05*	1.61 ± 0.15	0.022 ± 0.003	49.2 ± 6.7	5	7.00 ± 0.10*	3

pK_i_ values were calculated from inhibition of AV039 (50 nM) or XAC-*S*-ser-*S*-tyr-X-BY630 (25 nM) binding in Nluc-A_3_AR HEK293 cells measured by NanoBRET. The kinetic parameters, *k*_on_, *k*_off_ and pK_D_ values, were obtained by monitoring (via NanoBRET) the association of 40 nM AV039 or 20 nM XAC-*S*-ser-*S*-tyr-X-BY630 in the presence of various concentrations of unlabelled ligands on HEK293 Nluc-A_3_AR cells at 37 °C. The residence time (RT) was calculated as the reciprocal of the *k*_off_ values from each individual experiment. All values represent mean ± SEM from *n* separate experiments performed in triplicate

*p < 0.05, kinetic pK_D_ versus equilibrium competition pK_i_ values according to unpaired Student’s *t* test

**Fig. 7:**
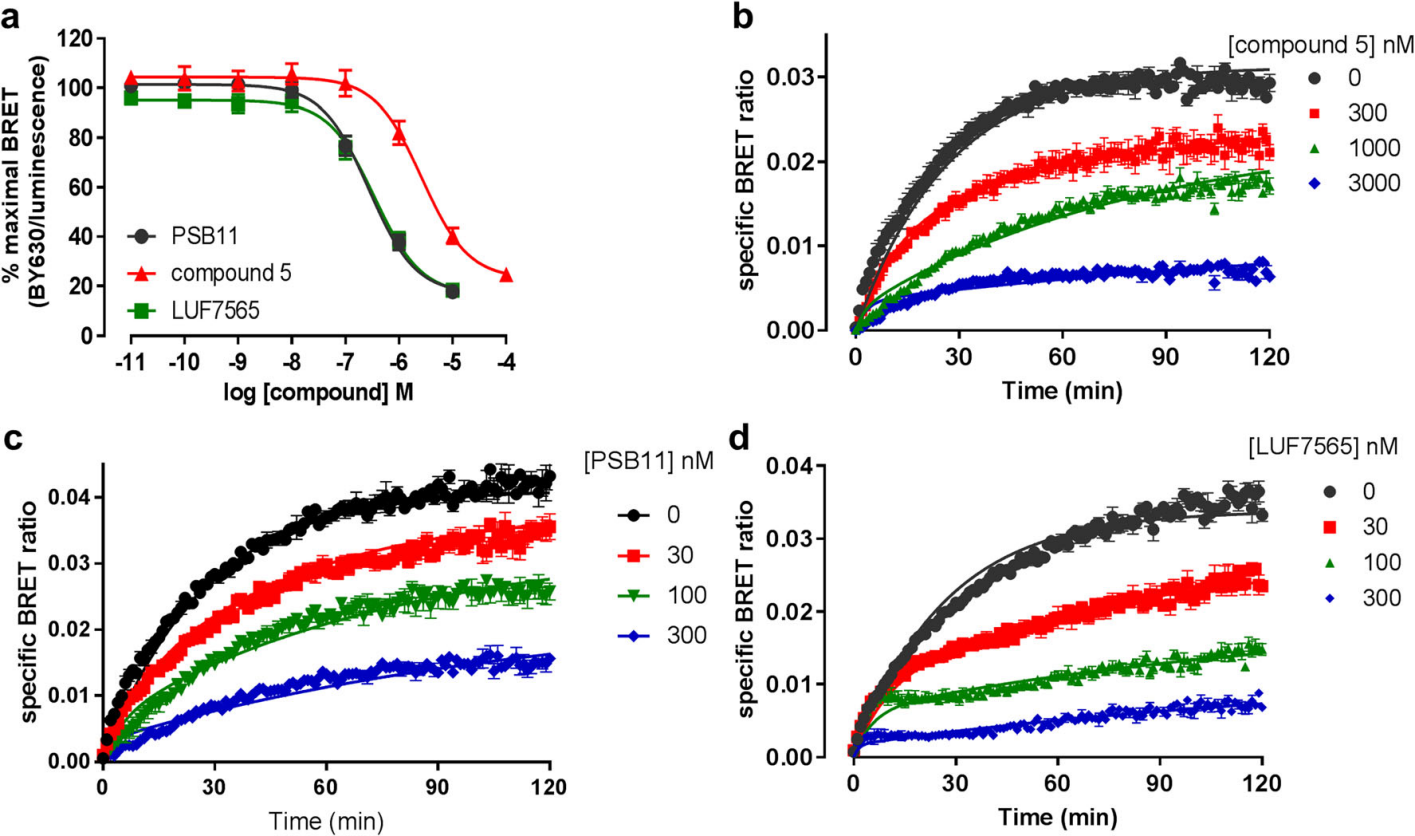
Characterisation of the effect of unlabelled ligands on the binding of XAC-*S*-ser-*S*-tyr-X-BY630 to Nluc-A_3_AR using NanoBRET. **a** Nluc-A_3_AR cells were treated with 40 nM XAC-*S*-ser-*S*-tyr-X-BY630 and increasing concentrations of PSB-11 (black circles), compound 5 (red triangles) and LUF7565 (green squares) for 3 h at 37 °C and competition binding curves generated. Data were normalised to the maximal BRET signal in each experiment. Data points are combined mean ± SEM from five (PSB-11) or six (compound 5 and LUF7565) separate experiments performed in triplicate. **b**–**d** The association of 20 nM XAC-*S*-ser-*S*-tyr-X-BY630 on HEK293 cells expressing human Nluc-A_3_AR at 37 °C was monitored at the indicated times by NanoBRET in either absence (control, black circles) or presence of various concentrations of compound 5 (**b**), PSB 11 (**c**) and LUF7565 (**d**). The data shown are representative examples from four (**b**, **c**) or five (**d**) independent experiments performed in triplicate, and the depicted data points represent the mean ± SEM of the triplicates

As XAC-*S*-ser-*S*-tyr-X-BY630 was found to have a considerably longer RT than AV039 and [^3^H]PSB-11, it was used to investigate the effect of the RT and binding kinetic parameters of the labelled probe on the measured kinetic parameters of unlabelled ligands. Firstly, it was confirmed that PSB-11, compound 5 and LUF7565 inhibited the specific XAC-*S*-ser-*S*-tyr-X-BY630 binding in a concentration-dependent manner in Nluc-A_3_AR HEK 293 cells (Fig. 7a; Table 3). It was noted that the affinity obtained for all the compounds was around half a log unit lower than the pK_i_ value obtained with AV039, but exhibited the same trend, with PSB-11 displaying the highest affinity at A_3_AR and compound 5 the least potent.

The concentration-dependent decrease in the association binding of XAC-*S*-ser-*S*-tyr-X-BY630 induced by the addition of different concentrations of PSB-11, compound 5 and LUF7565 allowed us to determine the association and dissociation rates of these unlabelled compounds when using a long RT compound as the probe. The competition association curves obtained in the presence of all three unlabelled ligands showed a continuous increase in XAC-*S*-ser-*S*-tyr-X-BY630 signal parallel to that observed with the fluorescent ligand only (Fig. 7b–d). Only a small initial overshoot in binding was observed in the presence of 100 and 300 nM of LUF7565. The kinetic parameters obtained for all ligands are presented in Table 3. The kinetic pK_D_ for compound 5 and PSB-11 was similar to that obtained at equilibrium, while LUF7565’s kinetic pK_D_ was slightly higher than the equilibrium pK_i_. In comparison to the kinetic parameters obtained with AV039, the *k*_on_ values were lower when using XAC-*S*-ser-*S*-tyr-X-BY630 as the probe for all three unlabelled compounds. For PSB-11 and compound 5, an increase in RT was observed with XAC-*S*-ser-*S*-tyr-X-BY630 in comparison to that with AV039, whereas a decrease in the RT of LUF7565 was seen.

## Discussion

It is becoming increasingly clear that a ligand’s binding kinetics are a crucial part of its pharmacology which can play a role in its success in the clinic [16, 18, 20]. In this study, we set out to develop an assay to determine the kinetic parameters of molecules binding to the A_3_AR using NanoBRET in living cells and at physiological temperatures. We also compared the values obtained to those obtained in a more classical radioligand binding assay that used cell membrane preparations and was performed at much lower temperatures (10 °C). We found that kinetic parameters of unlabelled ligands obtained in both assays were comparable when using a labelled probe with similar kinetics (AV039 versus [^3^H]PSB-11). Moreover, when a long RT probe (XAC-*S*-ser-*S*-tyr-X-BY630) was used, it was difficult to resolve the differences in kinetic parameters of a fast and slow unlabelled compound. This indicates that the kinetic profile of the labelled probe can influence the range of *k*_on_ and *k*_off_ values that can be accurately determined.

For many years, the toolbox available for studying binding kinetics at GPCRs was limited to the use of radioligand binding assays [14]. With the increasing range of fluorescent ligands available for the study of GPCRs alongside more suitable proteins for use in energy transfer techniques (such as NanoLuc and SNAP), there has been increased interest in adapting these techniques to measure ligand-binding kinetics [26]. Here, we have developed a BRET-based kinetic assay for the A_3_AR. This is particularly important for the A_3_AR, as historically there has only been a radiolabelled agonist commercially available, and radiolabelled antagonists, such as the one used in this study, have to be custom synthesised [35]. The A_3_AR offered a unique opportunity for this since multiple fluorescent ligands based on different parent molecules have been described in the literature and one of these ligands is commercially available [32–34]. Three of these fluorescent ligands measured in this study were based on the non-selective adenosine receptor antagonist XAC. Two of these ligands, CA200645 and XAC-*S*-ser-*S*-tyr-X-BYFL, were found to have similar *k*_on_ and *k*_off_ rate constants, whereas XAC-*S*-ser-*S*-tyr-X-BY630 had a similar value for *k*_on_ but a much slower *k*_off_ rate constant. In the development of fluorescent ligands with improved imaging properties, incorporation of a peptide linker between the pharmacophore and fluorophore was found to prevent the ligand from crossing the cell membrane in a non-specific manner [29, 33]. Molecular modelling has suggested that this linker region makes additional contacts at the top of the transmembrane domains of the receptor and that the fluorophore is buried within the lipid environment of the plasma membrane [33]. This has also been observed in molecular modelling of fluorescent ligands binding to the histamine H_1_ receptor [29]. Other studies that have developed fluorescent ligands have also found that the fluorophore can influence the affinity of the resulting molecule [39, 40]. It is therefore clear that the fluorophore portion of fluorescent ligand is not a passive part of the molecule but can influence both the affinity and binding kinetics. The chemical structures of XAC-*S*-ser-*S*-tyr-X-BY630 and XAC-*S*-ser-*S*-tyr-X-BYFL are very similar and only differ in the structure of the fluorophore. It has previously been shown with the β_2_-adrenoceptor that the interaction of ligands with the membrane influences the observed association rates [41]. The interaction of a ligand with a membrane can create a micro-environment which can alter the observed kinetic profiles [42]. It is possible that the BODIPY fluorophores interact differently with the cell membrane to increase the RT of XAC-*S*-ser-*S*-tyr-X-BY630 compared to XAC-*S*-ser-*S*-tyr-X-BYFL. It is worth noting, however, that the mere presence of a BY630 fluorophore is not a determinant for a longer RT compound since both CA200645 and AV039 contain the same fluorophore.

The availability of a radiolabelled antagonist, [^3^H]PSB-11, allowed us to compare the kinetic parameters of unlabelled ligands, measured using either long or short RT fluorescent ligand in the NanoBRET assay, with those obtained in a more traditional radioligand binding assay. We found that the *k*_on_ rate constants measured with all three unlabelled ligands were all within a similar range (Table 2) although the rank order within data sets was not the same. For example, LUF7565 was found to have the fastest association rate when either [^3^H]PSB-11 or XAC-*S*-ser-*S*-tyr-X-BY630 were used as the probe, whereas LUF7565 had the slowest association rate when measured against AV039. Although here we have only compared the kinetic parameters of three compounds, this is similar to previous studies comparing the kinetic parameters of GnRH ligands determined using radioligand binding and time-resolved FRET (TR-FRET) where no correlation between association rates was found in the two assay setups [43].

In comparison to the tight range of *k*_on_ values, there was a wider range of measured *k*_off_ values. For both [^3^H]PSB-11 and AV039, there was between a 30- and 94-fold difference, respectively, in the *k*_off_ values measured for compound 5 and LUF7565, with LUF7565 displaying the slowest *k*_off_ of the measured unlabelled compounds using both probes. This indicates that for both these probes, a wide range of kinetic parameters can be measured. As seen in Fig. 7, the profile of the competition association curves with each of the three unlabelled compounds was comparable, and this is reflected in the similarities in the kinetic parameters obtained. To date, LUF7565 is one of the longest residence time unlabelled ligands reported at A_3_AR, and it may be that XAC-*S*-ser-*S*-tyr-X-BY630 will be a useful tool to find even longer RT compounds at this receptor.

Although a similar trend was seen for the *k*_off_ values determined using [^3^H]PSB-11 and AV039, 10-fold higher *k*_off_ values were determined for PSB-11 and compound 5 when using AV039, leading to compounds with apparently shorter RT. There are many differences in setup of the two assays which could lead to these differences and need to be kept in mind when comparing the data sets. One of the main differences is that the radioligand binding assay uses membrane preparations, whereas the BRET assays are performed on intact cells. The intact cell environment preserves all of the intracellular proteins that a GPCR can interact with, and these are known to be able to stabilise different conformations of the receptor which in turn may affect the binding kinetics of the ligands [44, 45]. As these interacting proteins include G proteins and arrestins, preservation of the intact cellular environment allows downstream signalling and internalisation to still occur. It is unlikely that internalisation plays a role in this study since XAC-*S*-ser-*S*-tyr-X-BY630 and AV039 have previously been shown to be competitive antagonists, and in confocal imaging studies, there was no indication that these ligands caused internalisation of the A_3_AR [33, 34]. Although it appears that these fluorescent ligands do not cause internalisation of the receptor, it is possible that the antagonist bound form of the receptor may interact with different adaptor proteins and this may underpin some of the differences observed. Differences in binding kinetics have also been observed previously when comparing measurements from membrane preparations and whole cells at other GPCRs including the dopamine D_2L_ receptor [46] and histamine H_1_ receptor [29]. For the D_2L_ receptor, it was suggested that differences in dissociation rates are a reflection of how the ligand interacts with the cell membrane [46]. Apart from receptor environment, the two assay setups were performed at different temperatures—radioligand binding at 10 °C and BRET at 37 °C—and it can be assumed from basic thermodynamic principles that both association and dissociation rates will increase with temperature. Due to the rapid kinetics of [^3^H]PSB-11, the competition association assay is challenging to perform at higher temperature [30], and since the plate reader used for the live cell assay lacks the capacity to be actively cooled, we are unable at the present time to directly test this. An increase in association and dissociation rate with temperature has been observed for the histamine H_1_ receptor [47] and the prolactin receptor [48].

In summary, this study has demonstrated that a NanoBRET-based assay can be applied to measure ligand-binding kinetics at the A_3_AR in intact living cells at physiological temperatures. The data shown here do, however, indicate that care needs to be taken when selecting a probe with the appropriate kinetics for the study of unlabelled ligands with different kinetic profiles. Thus, probes with very slow kinetics may be problematic for the determination of the kinetic parameters of unlabelled ligands with rapid kinetic properties. As the range of fluorescent ligands for GPCRs continues to expand [49], their use in NanoBRET-based assays should provide the required diversity of kinetic properties to evaluate the kinetic profiles of the broadest range of unlabelled ligands in a physiologically relevant setting.

## References

[1] Elliott MR, Chekeni FB, Trampont PC, Lazarowski ER, Kadl A, Walk SF, Park D, Woodson RI, Ostankovich M, Sharma P, Lysiak JJ, Harden TK, Leitinger N, Ravichandran KS (2009). Nucleotides released by apoptotic cells act as a find-me signal to promote phagocytic clearance. Nature.

[2] Okada SF, Nicholas RA, Kreda SM, Lazarowski ER, Boucher RC (2006). Physiological regulation of ATP release at the apical surface of human airway epithelia. J Biol Chem.

[3] Picher M, Burch LH, Hirsh AJ, Spychala J, Boucher RC (2003). Ecto 5'-nucleotidase and nonspecific alkaline phosphatase. Two AMP-hydrolyzing ectoenzymes with distinct roles in human airways. J Biol Chem.

[4] Fredholm BB, IJzerman AP, Jacobson KA, Linden J, Müller CE (2011). International Union of Basic and Clinical Pharmacology. LXXXI. Nomenclature and classification of adenosine receptors: an update. Pharmacol Rev.

[5] Jacobson KA, Merighi S, Varani K, Borea PA, Baraldi S, Aghazadeh Tabrizi M, Romagnoli R, Baraldi PG, Ciancetta A, Tosh DK, Gao ZG, Gessi S (2017). A3 adenosine receptors as modulators of inflammation: from medicinal chemistry to therapy. Med Res Rev.

[6] Germack R, Dickenson JM (2004). Characterization of ERK1/2 signalling pathways induced by adenosine receptor subtypes in newborn rat cardiomyocytes. Br J Pharmacol.

[7] McIntosh VJ, Lasley RD (2012). Adenosine receptor-mediated cardioprotection: are all 4 subtypes required or redundant?. J Cardiovasc Pharmacol Ther.

[8] Pugliese AM, Coppi E, Volpini R, Cristalli G, Corradetti R, Jeong LS, Jacobson KA, Pedata F (2007). Role of adenosine A3 receptors on CA1 hippocampal neurotransmission during oxygen-glucose deprivation episodes of different duration. Biochem Pharmacol.

[9] Rivera-Oliver M, Diaz-Rios M (2014). Using caffeine and other adenosine receptor antagonists and agonists as therapeutic tools against neurodegenerative diseases: a review. Life Sci.

[10] Fishman P, Bar-Yehuda S, Barer F, Madi L, Multani AS, Pathak S (2001). The A3 adenosine receptor as a new target for cancer therapy and chemoprotection. Exp Cell Res.

[11] Fishman P, Bar-Yehuda S, Liang BT, Jacobson KA (2012). Pharmacological and therapeutic effects of A3 adenosine receptor agonists. Drug Discov Today.

[12] Hauser RA, Stocchi F, Rascol O, Huyck SB, Capece R, Ho TW, Sklar P, Lines C, Michelson D, Hewitt D (2015). Preladenant as an adjunctive therapy with levodopa in Parkinson disease: two randomized clinical trials and lessons learned. JAMA Neurol.

[13] Waring MJ, Arrowsmith J, Leach AR, Leeson PD, Mandrell S, Owen RM, Pairaudeau G, Pennie WD, Pickett SD, Wang J, Wallace O, Weir A (2015). An analysis of the attrition of drug candidates from four major pharmaceutical companies. Nat Rev Drug Discov.

[14] Guo D, Hillger JM, AP IJ, Heitman LH (2014). Drug-target residence time—a case for G protein-coupled receptors. Med Res Rev.

[15] Vauquelin G (2016). Effects of target binding kinetics on in vivo drug efficacy: koff , kon and rebinding. Br J Pharmacol.

[16] Schuetz DA, de Witte WEA, Wong YC, Knasmueller B, Richter L, Kokh DB, Sadiq SK, Bosma R, Nederpelt I, Heitman LH, Segala E, Amaral M, Guo D, Andres D, Georgi V, Stoddart LA, Hill S, Cooke RM, De Graaf C, Leurs R, Frech M, Wade RC, de Lange ECM, AP IJ, Muller-Fahrnow A, Ecker GF (2017). Kinetics for drug discovery: an industry-driven effort to target drug residence time. Drug Discov Today.

[17] Swinney DC, Haubrich BA, Van Liefde I, Vauquelin G (2015). The role of binding kinetics in GPCR drug discovery. Curr Top Med Chem.

[18] Copeland RA, Pompliano DL, Meek TD (2006). Drug-target residence time and its implications for lead optimization. Nat Rev Drug Discov.

[19] Beeh KM, Westerman J, Kirsten AM, Hebert J, Gronke L, Hamilton A, Tetzlaff K, Derom E (2015). The 24-h lung-function profile of once-daily tiotropium and olodaterol fixed-dose combination in chronic obstructive pulmonary disease. Pulm Pharmacol Ther.

[20] Tautermann CS (2016). Impact, determination and prediction of drug-receptor residence times for GPCRs. Curr Opin Pharmacol.

[21] Vauquelin G (2016). Cell membranes... and how long drugs may exert beneficial pharmacological activity in vivo. Br J Clin Pharmacol.

[22] Kapur S, Seeman P (2001). Does fast dissociation from the dopamine d(2) receptor explain the action of atypical antipsychotics?: a new hypothesis. Am J Psychiatry.

[23] Segala E, Errey JC, Fiez-Vandal C, Zhukov A, Cooke RM (2015). Biosensor-based affinities and binding kinetics of small molecule antagonists to the adenosine A(2A) receptor reconstituted in HDL like particles. FEBS Lett.

[24] Aristotelous T, Ahn S, Shukla AK, Gawron S, Sassano MF, Kahsai AW, Wingler LM, Zhu X, Tripathi-Shukla P, Huang XP, Riley J, Besnard J, Read KD, Roth BL, Gilbert IH, Hopkins AL, Lefkowitz RJ, Navratilova I (2013). Discovery of beta2 adrenergic receptor ligands using biosensor fragment screening of tagged wild-type receptor. ACS Med Chem Lett.

[25] Hoffmann C, Castro M, Rinken A, Leurs R, Hill SJ, Vischer HF (2015). Ligand residence time at G-protein-coupled receptors-why we should take our time to study it. Mol Pharmacol.

[26] Stoddart LA, White CW, Nguyen K, Hill SJ, Pfleger KD (2016). Fluorescence- and bioluminescence-based approaches to study GPCR ligand binding. Br J Pharmacol.

[27] Motulsky HJ, Mahan LC (1984). The kinetics of competitive radioligand binding predicted by the law of mass action. Mol Pharmacol.

[28] Schiele F, Ayaz P, Fernandez-Montalvan A (2014). A universal homogeneous assay for high-throughput determination of binding kinetics. Anal Biochem.

[29] Stoddart LA, Vernall AJ, Bouzo-Lorenzo M, Bosma R, Kooistra AJ, de Graaf C, Vischer HF, Leurs R, Briddon SJ, Kellam B, Hill SJ (2018). Development of novel fluorescent histamine H1-receptor antagonists to study ligand-binding kinetics in living cells. Sci Rep.

[30] Xia L, Burger WAC, van Veldhoven JPD, Kuiper BJ, van Duijl TT, Lenselink EB, Paasman E, Heitman LH, AP IJ (2017). Structure-affinity relationships and structure-kinetics relationships of pyrido[2,1-f]purine-2,4-dione derivatives as human adenosine A3 receptor antagonists. J Med Chem.

[31] Zeilinger M, Pichler F, Nics L, Wadsak W, Spreitzer H, Hacker M, Mitterhauser M (2017). New approaches for the reliable in vitro assessment of binding affinity based on high-resolution real-time data acquisition of radioligand-receptor binding kinetics. EJNMMI Res.

[32] Stoddart LA, Vernall AJ, Denman JL, Briddon SJ, Kellam B, Hill SJ (2012). Fragment screening at adenosine-A(3) receptors in living cells using a fluorescence-based binding assay. Chem Biol.

[33] Vernall AJ, Stoddart LA, Briddon SJ, Ng HW, Laughton CA, Doughty SW, Hill SJ, Kellam B (2013). Conversion of a non-selective adenosine receptor antagonist into A3-selective high affinity fluorescent probes using peptide-based linkers. Org Biomol Chem.

[34] Vernall AJ, Stoddart LA, Briddon SJ, Hill SJ, Kellam B (2012). Highly potent and selective fluorescent antagonists of the human adenosine a(3) receptor based on the 1,2,4-triazolo 4,3-a quinoxalin-1-one scaffold. J Med Chem.

[35] Muller CE, Diekmann M, Thorand M, Ozola V (2002). [(3)H]8-Ethyl-4-methyl-2-phenyl-(8R)-4,5,7,8-tetrahydro-1H-imidazo[2,1-i]-purin-5 -one ([(3)H]PSB-11), a novel high-affinity antagonist radioligand for human A(3) adenosine receptors. Bioorg Med Chem Lett.

[36] Priego EM, von Frijtag Drabbe Kuenzel J, AP IJ, Camarasa MJ, Perez-Perez MJ (2002). Pyrido[2,1-f]purine-2,4-dione derivatives as a novel class of highly potent human A(3) adenosine receptor antagonists. J Med Chem.

[37] Smith PK, Krohn RI, Hermanson GT, Mallia AK, Gartner FH, Provenzano MD, Fujimoto EK, Goeke NM, Olson BJ, Klenk DC (1985). Measurement of protein using bicinchoninic acid. Anal Biochem.

[38] Stoddart LA, Johnstone EK, Wheal AJ, Goulding J, Robers MB, Machleidt T, Wood KV, Hill SJ, Pfleger KD (2015). Application of BRET to monitor ligand binding to GPCRs. Nat Methods.

[39] Baker JG, Middleton R, Adams L, May LT, Briddon SJ, Kellam B, Hill SJ (2010). Influence of fluorophore and linker composition on the pharmacology of fluorescent adenosine A(1) receptor ligands. Br J Pharmacol.

[40] Daval SB, Valant C, Bonnet D, Kellenberger E, Hibert M, Galzi JL, Ilien B (2012). Fluorescent derivatives of AC-42 to probe bitopic orthosteric/allosteric binding mechanisms on muscarinic M1 receptors. J Med Chem.

[41] Sykes DA, Parry C, Reilly J, Wright P, Fairhurst RA, Charlton SJ (2014). Observed drug-receptor association rates are governed by membrane affinity: the importance of establishing “micro-pharmacokinetic/pharmacodynamic relationships” at the beta2-adrenoceptor. Mol Pharmacol.

[42] Vauquelin G, Charlton SJ (2010). Long-lasting target binding and rebinding as mechanisms to prolong in vivo drug action. Br J Pharmacol.

[43] Nederpelt I, Georgi V, Schiele F, Nowak-Reppel K, Fernandez-Montalvan AE, AP IJ, Heitman LH (2016). Characterization of 12 GnRH peptide agonists—a kinetic perspective. Br J Pharmacol.

[44] Vanderheyden PML, Benachour N (2017). Influence of the cellular environment on ligand binding kinetics at membrane-bound targets. Bioorg Med Chem Lett.

[45] McRobb FM, Negri A, Beuming T, Sherman W (2016). Molecular dynamics techniques for modeling G protein-coupled receptors. Curr Opin Pharmacol.

[46] Packeu A, De Backer JP, Van Liefde I, Vanderheyden PM, Vauquelin G (2008). Antagonist-radioligand binding to D2L-receptors in intact cells. Biochem Pharmacol.

[47] Treherne JM, Young JM (1988). Temperature-dependence of the kinetics of the binding of [3H]-(+)-N-methyl-4-methyldiphenhydramine to the histamine H1-receptor: comparison with the kinetics of [3H]-mepyramine. Br J Pharmacol.

[48] Sakai S (1991). Effect of hormones on dissociation of prolactin from the rabbit mammary gland prolactin receptor. Biochem J.

[49] Vernall AJ, Hill SJ, Kellam B (2014). The evolving small-molecule fluorescent-conjugate toolbox for class A GPCRs. Br J Pharmacol.

